# Retraction: Recursive fury: conspiracist ideation in the blogosphere in response to research on conspiracist ideation

**DOI:** 10.3389/fpsyg.2014.00293

**Published:** 2014-03-27

**Authors:** Frontiers in Psychology Editorial Office

In the light of a small number of complaints received following publication of the original research article cited above, Frontiers carried out a detailed investigation of the academic, ethical, and legal aspects of the work. This investigation did not identify any issues with the academic and ethical aspects of the study. It did, however, determine that the legal context is insufficiently clear and therefore Frontiers wishes to retract the published article. The authors understand this decision, while they stand by their article and regret the limitations on academic freedom which can be caused by legal factors.

